# Plasma proteome atlas for differentiating tumor stage and post-surgical prognosis of hepatocellular carcinoma and cholangiocarcinoma

**DOI:** 10.1371/journal.pone.0238251

**Published:** 2020-08-26

**Authors:** Ting-Tsung Chang, Cheng-Hsun Ho

**Affiliations:** 1 Department of Internal Medicine, National Cheng Kung University Hospital, College of Medicine, National Cheng Kung University, Tainan, Taiwan; 2 Department of Medical Laboratory Science, College of Medicine, I-Shou University, Kaohsiung City, Taiwan; University of Navarra School of Medicine and Center for Applied Medical Research (CIMA), SPAIN

## Abstract

Although mass spectrometry-based plasma proteomics enables sensitive and large-scale discovery and validation of biomarkers for various diseases, its integrative application to hepatocellular carcinoma (HCC) and cholangiocarcinoma (CCA) is not well investigated. Therefore, we analyzed albumin- and immunoglobulin G-depleted plasma samples from 148 and 60 patients with HCC and CCA, respectively, using liquid chromatography-tandem mass spectrometry. The algorithm used to measure the content of each protein was the percentage of exponentially modified protein abundance index. From 5320 proteins assayed in plasma, 53 and 25 biomarker candidates were identified for HCC and CCA, respectively. The abundance of six and two HCC markers particularly protruded in stage II and III, respectively, whereas plasma serine protease inhibitor was the sole marker the level of which steadily decreased with CCA progression. From a prognostic facet, we showed candidate markers and their cutoff levels for evaluating probability of tumor recurrence and patient survival period. Combination Kaplan-Meier models showed that HCC stage III or IV and both the content of alpha-2-HS-glycoprotein and apolipoprotein CIII <0.2% exhibited the poorest post-surgical recurrence-free and overall survivals. Furthermore, the content of afamin ≥0.2% played a significant role on the poor prognosis in patients with CCA. Our findings, taken together, characterized novel plasma biomarker signatures in dissecting tumor stages and post-surgical outcomes of HCC and CCA.

## Introduction

Hepatocellular carcinoma (HCC) and cholangiocarcinoma (CCA; also known as bile duct cancer) are the two most common types of hepatobiliary malignancies, arising from neoplasms of hepatocytes and cholangiocytes, respectively. Hepatobiliary cancer is the fifth most common type of cancer and the third most common cause of cancer-related deaths worldwide. Approximately 700,000 new cases of HCC are reported annually on a global scale, of which hepatitis B or C virus-infected cases account for more than 75% [1]. CCA incidence has risen over the last two decades, particularly in Southeast Asia, with an estimated 130,000 new cases per year. Depending on the tumor stage, hepatic functional reserve, and performance status of patients, the treatment options for HCC include radiofrequency ablation, transarterial chemoembolization, radioembolization, multikinase inhibitors, hepatic resection, and liver transplantation [2]. The 5-year survival rates of HCC at early and late diagnoses are approximately 30% and lower than 15%, respectively. Unlike HCC, therapeutic choices for CCA are limited because it is strongly resistant to chemotherapy. The curative options are surgery and liver transplantation in its early stage. However, CCA is difficult to diagnose, aggressive, and heterogenous; therefore, less than one-third of such cancers are unresectable or metastasized at diagnosis. With this, the remnant treatments available are systemic or palliative therapy, leading to a 5-year mortality rate of higher than 90% [3,4].

Mass spectrometry-based proteomics in liquid biopsies is one of the most powerful platforms to noninvasively determine protein biomarkers of various diseases because it is easy to access, has high sensitivity to identify targets at a very low abundance within complex mixtures, and can detect negligible differences in expression levels. The hepatobiliary system is the major contributor to the plasma protein pool; hence, plasma proteomics should be leveraged in the diagnosis, elucidation of the oncological processes, and prognosis of HCC and CCA. Early diagnosis of tumor greatly improves the curative frequency and medical outcomes of patients, and plasma proteome analysis for HCC and CCA will not only solve technical problems on tumor staging, such as insufficient resolution in cancer screening and sampling bias in liver biopsy, but also facilitate longitudinal tracking of the disease status [5]. For this purpose, we performed a label-free, quantitative proteomics study to search plasma protein markers specific to HCC and CCA, addressed their expression patterns in different tumor stages, and evaluated their applicability to tumor recurrence and patients’ survival.

## Materials and methods

### Patients and study design

The Institutional Review Board of National Cheng Kung University Hospital (NCKUH) approved our study (approval numbers: B-ER-103-133 and B-ER-105-098), which was conducted following the guidelines of the Declaration of Helsinki. We collected the plasma samples, clinical data, and laboratory data of patients with HCC (n = 148) and CCA (n = 60) who underwent surgical resection from the Tissue Bank, Research Center of Clinical Medicine, NCKUH. Medical records of the patients were accessed from December 2002 to March 2014, and all data were fully anonymized before obtaining access. All the patients could not be identified. We also enrolled 95 control participants who were negative for hepatobiliary diseases from the Health Examination Center of NCKUH and obtained written informed consent from them. All plasma samples were stored at -80°C until use. We followed the TNM classification guidelines given in the eighth edition of the American Joint Committee on Cancer Staging Manual for staging HCC or CCA.

### Sample preparation and liquid chromatography-tandem mass spectrometry (LC-MS/MS) analysis

Five microliters of plasma were diluted with phosphate-buffered saline and incubated with CaptureSelect Human Albumin Affinity Matrix (Thermo Fisher Scientific, Waltham, MA, USA) and Protein G-sepharose beads (GE Healthcare, Piscataway, NJ, USA) to remove albumin and immunoglobulin (Ig) G. Proteins not bound to the beads were harvested, denatured using 10% sodium dodecyl sulfate plus 10 mM dithiothreitol at 95°C for 10 minutes, and alkylated with 10 mM iodoacetamide at 37°C in dark for 1 hour. The proteins were then cleaned and concentrated using Amicon Ultra-0.5 mL centrifugal filters (molecular weight cutoff: 3,000 Da) device (Merck Millipore, Darmstadt, Germany) and digested at 37°C overnight using sequencing grade trypsin (Promega, Fitchburg, WI, USA) in 10 mM ammonium bicarbonate in an enzyme-to-substrate ratio of 1:50. We followed the procedure of liquid chromatography-tandem mass spectrometry (LC-MS/MS) analysis outlined in our previous study [6]. A rapid separation liquid chromatography system (Ultimate 3000; Dionex, Santa Clara, CA, USA) equipped with a C18 column (Acclaim PepMap RSLC, 75μm × 150 mm, 2 μm, 100 Å) was coupled to a Q Exactive Orbitrap mass spectrometer (Thermo Fisher Scientific). Mobile phase A and B consisted of 0.1% fluoroacetic acid and 0.1% fluoroacetic acid in 95% acetonitrile, respectively. The gradient comprised a linear increase from 1% to 25% B over 45 minutes, a linear increase from 25% to 60% B over 10 minutes, and finally, isocratic elution at 80% B for 10 minutes at 250 nL/minute for separation. A full MS spectrum (*m/z* 300–2000) with a mass resolution of 140,000 was acquired, followed by 10 sequential collision-induced dissociation-MS^2^ scans using the mass spectrometer in data-dependent mode.

### Protein identification and quantification

Raw data of LC-MS/MS were processed into peak lists by Proteome Discoverer 1.4 for Mascot database (version 2.4.1, Matrix Science Ltd., London, UK) searched against the Swiss-Prot_2015_07 database. Parameters were set as follows: enzyme, trypsin; missed cleavages, 1; peptide mass tolerance, 10 ppm; fragment mass tolerance, 0.05 Da; fixed modification, carbamidomethyl (C). The exponentially modified protein abundance index (emPAI) was used to calculate the number of sequenced peptides per protein [7]. The percentage of each emPAI from the summation of all the emPAI values for the identified proteins was leveraged as the algorithm to quantify the content of each protein [8]. All identified targets were included in the calculation of emPAI% of each protein to avoid creating a bias when measuring the content of each protein. However, biomarker candidates shown a protein score <30 were removed from the list of marker candidates to reduce a false identification. All analysts were blinded to any information about the subjects.

### Statistical analysis

Statistical analyses were performed using PASW Statistics for Windows (version 18.0; SPSS Inc., Chicago, IL, USA). Differential proteins in HCC or CCA were identified using receiver operator characteristic curves (the area under the ROC curve > 0.7 and *P* < 0.00001). Continuous and nominal variables in different tumor stages were compared using Kruskal-Wallis tests and Pearson Chi-square tests, respectively.

Venn diagrams were obtained using InteractiVenn (http://bioinfogp.cnb.csic.es/tools/venny/) [9]. The emPAI% values of protein markers in different tumor stages were compared using one-way analysis of variance with Scheffé posterior comparison. Protein levels between advanced-stage and non-advanced-stage HCC and CCA as well as between metastatic and non-metastatic HCC and CCA were compared using Mann–Whitney *U* tests. The significance of protein biomarkers on recurrence-free and overall survivals of the patients was assessed using Kaplan-Meier analyses in combination with log-rank tests. Proteins associated with post-surgical tumor recurrence and mortality were identified using stepwise Cox regression analyses. Both models with and without a post-univariate Bonferroni correction were shown. Significance was set as two-tailed *P* < 0.05.

## Results

### Characteristics of patients at different HCC and CCA stages

We used the American Joint Committee on Cancer staging system to classify the cancer stage of the patients. Of the 148 patients with HCC, 58, 55, 20, 5, 6, and 4 were in stage I, II, IIIA, IIIB, IIIC, and IV, respectively. Although they were at different stages of HCC, the patients demonstrated similar demographic, biochemical, and hematological data (Table 1). The median alanine aminotransferase and aspartate aminotransferase levels in the different tumor stages were below three and two times the upper limit of normal, respectively. Hepatitis B virus infection and liver cirrhosis were prevalent in more than 50% and more than 60% of patients with stage I-III HCC (Table 1). The proportion of cirrhotic patients increased from 45% in stage IIIA HCC to 100% in stage IIIB HCC (S1 Table). Furthermore, post-surgical HCC recurrence was observed in over 70% of these patients in five years. The 5-year survival rates declined in stages III and IV HCC as expected, with a 100% mortality rate in stages IIIC and IV.

**Table 1:** Characteristics of patients with different tumor stage of hepatocellular carcinoma and cholangiocarcinoma.

Variable	Stage I	Stage II	Stage III^†^	Stage IV^‡^	*P*-value
*Hepatocellular carcinoma*					
Number	58	55	31	4	
Male, n (%)	48 (82.8%)	39 (70.9%)	21 (67.7%)	2 (50.0%)	0.217
Age (years)	61 (23–86)	58 (39–82)	61 (44–84)	53 (45–65)	0.089
Alanine aminotransferase (U/L)	53 (13–432)	55 (10–365)	51 (13–436)	93 (28–306)	0.904
Aspartate aminotransferase (U/L)	52 (17–175)	56 (21–645)	50 (22–800)	58 (17–66)	0.727
Albumin (g/dL)	4.2 (2.6–5.0)	4.2 (1.8–5.1)	3.9 (1.8–4.8)	3.9 (2.3–4.0)	0.055
Alkaline phosphatase (U/L)*	80.5 (46–174)	96 (52–913)	118 (50–976)	77 (70–86)	0.050
Total bilirubin (mg/dL)	0.6 (0.2–1.5)	0.6 (0.2–1.7)	0.6 (0.3–7.0)	0.5 (0.3–0.7)	0.304
α-fetoprotein (ng/mL)*	12.2 (1.7–45182.0)	38.0 (1.4–4590.0)	403.4 (0.9–32420.0)	45.3 (1.9–88.6)	0.508
Red blood cell (10^6^/μL)	4.1 (2.4–5.5)	4.4 (2.8–6.1)	4.1 (2.9–4.8)	4.2 (3.8–4.5)	0.277
White blood cell (10^3^/μL)	5.7 (2.0–10.0)	5.7 (2.7–8.1)	6.0 (3.9–10.4)	8.3 (5.7–8.8)	0.093
Platelet (10^3^/μL)	164 (33–549)	152 (47–415)	197 (88–400)	194 (173–223)	0.075
Hepatitis B, n (%)	30 (51.7%)	32 (58.2%)	21 (67.7%)	1 (25.0%)	0.283
Hepatitis C, n (%)	23 (39.7%)	20 (36.4%)	5 (16.1%)	1 (25.0%)	0.131
Liver cirrhosis, n (%)	35 (60.3%)	36 (65.5%)	19 (61.3%)	0 (0.0%)	0.082
Follow-up 5-Year recurrence, n (%)	42 (72.4%)	39 (70.9%)	24 (77.4%)	2 (50.0%)	0.693
Follow-up 5-Year survivals, n (%)	27 (46.6)	25 (45.5%)	6 (19.4%)	0 (0.0%)	0.019
*Cholangiocarcinoma*					
Number	11	22	6	21	
Male, n (%)	5 (45.5%)	14 (63.6%)	1 (16.7%)	11 (52.4%)	0.223
Age (years)	56 (33–76)	65.5 (47–87)	63.5 (57–72)	67 (38–85)	0.456
Alanine aminotransferase (U/L)	35 (12–151)	38.5 (11–162)	39 (13–99)	35 (10–199)	0.916
Aspartate aminotransferase (U/L)	40 (24–231)	54.5 (19–211)	37.5 (28–101)	44 (17–125)	0.814
Albumin (g/dL)	4.4 (3.8–4.8)	4.4 (2.9–5.2)	4.0 (3.0–4.4)	4.1 (3.2–4.9)	0.076
Alkaline phosphatase (U/L)*	118 (25–741)	166 (59–786)	145.5 (26–657)	138 (56–444)	0.632
Total bilirubin (mg/dL)	0.6 (0.3–1.1)	0.7 (0.2–11.6)	0.7 (0.4–11.8)	0.6 (0.2–9.8)	0.718
Carbohydrate antigen 19–9 (U/mL)*	103.7 (7.3–5791.0)	264.2 (3.5–5206.0)	249.1 (17.5–10000.0)	195.8 (0.1–36622.0)	0.822
Red blood cell (10^6^/μL)	4.4 (3.4–5.1)	4.3 (3.0–5.6)	4.1 (3.7–4.5)	3.9 (2.6–5.0)	0.352
White blood cell (10^3^/μL)	6.9 (4.2–9.1)	5.9 (3.6–12.1)	8.2 (7.0–10.0)	7.3 (3.7–16.9)	0.026
Platelet (10^3^/μL)	180 (84–316)	210.5 (133–394)	267 (247–412)	215 (108–359)	0.057
Hepatitis B, n (%)	7 (63.6%)	5 (22.7%)	1 (16.7%)	6 (28.6%)	0.081
Hepatitis C, n (%)	2 (18.2%)	3 (13.6%)	0 (0.0%)	2 (9.5%)	0.700
Follow-up 5-Year recurrence, n (%)	4 (36.4%)	10 (45.5%)	4 (66.7%)	7 (33.3%)	0.494
Follow-up 5-Year survivals, n (%)	4 (36.4%)	8 (36.4%)	0 (0.0%)	1 (4.8%)	0.024

Data are numbers (percentages) or median values (minimum − maximum).

*contain missing values.

^†^HCC: 20 stage IIIA, 5 stage IIIB, and 6 stage IIIC.

^‡^HCC: 2 stage IVA and 2 stage IVB; CCA: 18 stage IVA and 3 stage IVB. Nominal values are compared using Pearson Chi-square tests. Continuous variables are compared using Kruskal-Wallis tests.

Among patients with CCA, 11, 22, 6, and 21 were in stages I, II, III, and IV, respectively (Table 1). No gender and age differences among patients in different tumor stages were found. Biochemical testing results and hepatitis B or C virus infection rate also did not alter the CCA progression. A slight increase in white blood cell count was seen in stage III CCA. A post-surgical recurrence rate of more than 30% was observed in each tumor stage of CCA. At the 5-year post-surgical follow-up, 100% and 95.2% mortality rates of patients in stages III and IV CCA, respectively, were seen.

### Plasma proteome profiles and protein markers for HCC and CCA

**Fig 1:**
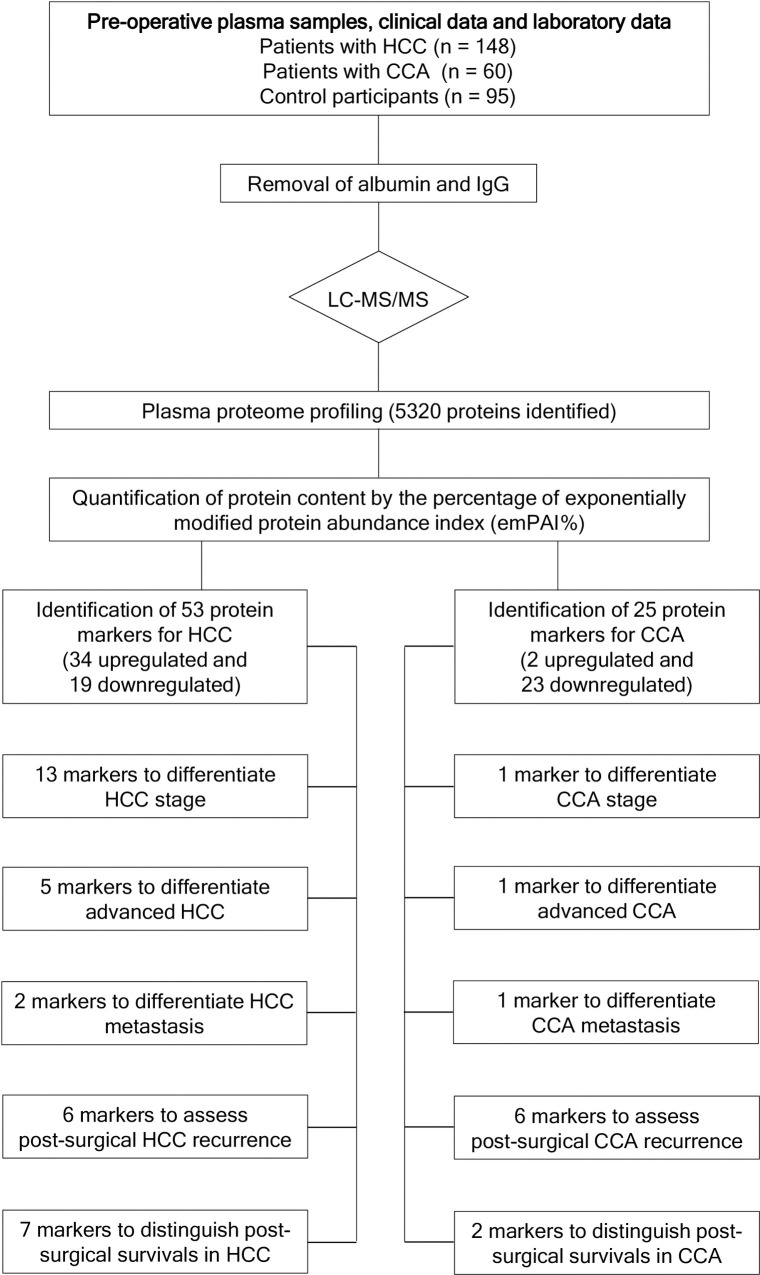
The flowchart of the study design. CCA, cholangiocarcinoma; IgG, immunoglobulin G; HCC, hepatocellular carcinoma; LC-MS/MS, liquid chromatography–tandem mass spectrometry.

**Fig 2:**
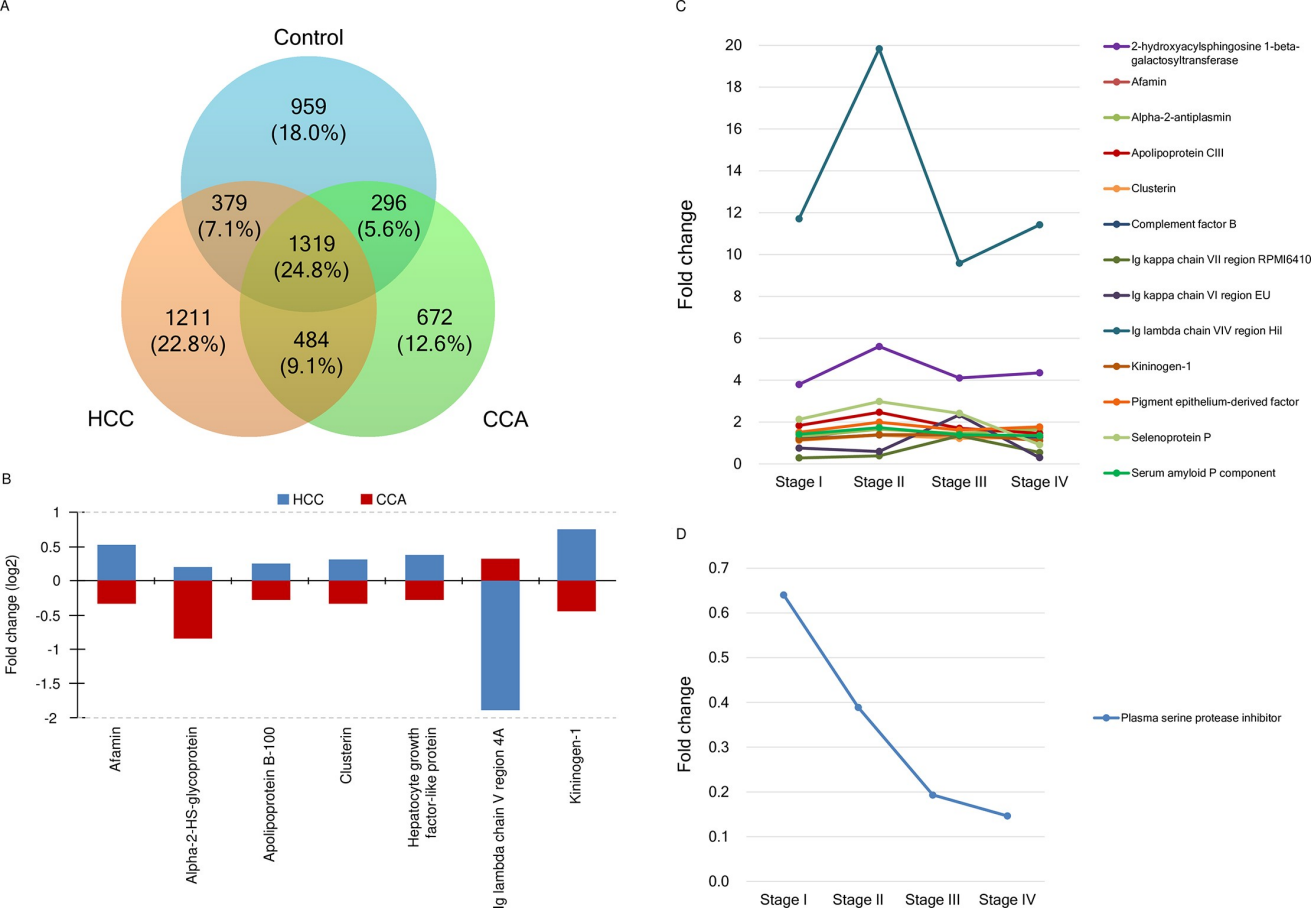
Identification of plasma protein markers for hepatocellular carcinoma (HCC) and cholangiocarcinoma (CCA). (A) Venn diagrams for plasma proteome in HCC, CCA, and control groups are shown. (B) Fold changes of co-differential proteins in HCC and CCA are shown in bar graphs. Fold changes of differential proteins of (C) HCC and (D) CCA in different stages are shown. The fold change of each protein was calculated using the mean percentage of exponentially modified protein abundance index (emPAI%) from the patients in normalization with the mean emPAI% in the controls.

A flowchart of this study was shown in Fig 1. A total of 5320 proteins were identified in the pre-operative, albumin- and IgG-depleted plasma samples of the subjects. The minimal protein score of identified protein was 13. Of 30 randomly selected plasma samples, the mean false discovery rate of protein identification was 1.35%. Our results demonstrated that 1319 proteins (24.8%) were common to all three groups, whereas 1211 (22.8%), 672 (12.6%), and 959 (18.0%) were unique to HCC, CCA, and controls, respectively (Fig 2A). The biomarker candidates for HCC (Table 2) and CCA (Table 3) were 53 (34 upregulated and 19 downregulated) and 25 (2 upregulated and 23 downregulated), respectively. Of these, 12 for HCC and 6 for CCA possessed both specificity and sensitivity of more than 70%. Comparisons of our HCC plasma biomarker candidates with 2 review articles regarding circulating or secretory HCC protein biomarkers were shown in S1 Fig. Few common HCC biomarkers were identified among each other. Our data showed that expressions of afamin, alpha-2-HS-glycoprotein, apolipoprotein B-100, clusterin, hepatocyte growth factor-like protein, and kininogen-1 were stimulated in HCC but repressed in CCA, while the expression of Ig lambda chain V region 4A was stimulated in CCA but repressed in HCC (Fig 2B). Levels of five biomarker candidates for HCC were higher in stage III and IV than in stage I and II and levels of fibrinogen gamma chain and selenoprotein P changed in metastatic HCC (S2 Fig). Moreover, plasma serine protease inhibitor was found to be downregulated in advanced and metastatic CCA.

**Table 2:** Differential plasma proteins for hepatocellular carcinoma (HCC).

Protein name	AUC	Standard error	Cut-off emPAI%	Sensitivity (%)	Specificity (%)
*Cell-cell interaction or adhesion*					
Thrombospondin 1 ↓	0.766	0.028	0.0065	68.4	79.7
*Coagulation*					
Coagulation factor XIII A chain ↑	0.715	0.030	0.0015	58.8	83.2
Fibrinogen alpha chain ↑	0.831	0.025	1.0315	99.3	63.2
Fibrinogen gamma chain ↑	0.779	0.029	1.1335	96.6	67.1
Kininogen-1 ↑	0.708	0.030	0.3175	75.0	60.0
Platelet factor 4 ↓	0.743	0.029	0.0535	48.4	100.0
*Complement-associated factors*					
Complement C5 ↑	0.744	0.029	0.1200	62.2	87.1
Complement component C7 ↑	0.732	0.028	0.0905	67.6	67.7
Complement factor B ↑	0.737	0.029	0.3410	67.6	72.9
Complement factor H-related protein 2 ↑	0.713	0.031	0.1405	58.8	83.9
*Transferase*					
2-hydroxyacylsphingosine 1-beta-galactosyltransferase ↑	0.811	0.026	0.0095	70.3	85.8
Transketolase-like protein 2 ↓	0.739	0.029	0.0025	62.6	84.5
*Immunoglobulin-related protein*					
Ig heavy chain VI region V35 ↑	0.706	0.030	0.0725	57.4	81.3
Ig heavy chain VII region ARH77 ↓	0.754	0.029	0.0380	66.5	86.5
Ig heavy chain VIII region 23 ↓	0.803	0.027	0.1395	76.8	85.8
Ig heavy chain VIII region CAM ↓	0.733	0.032	0.0880	87.1	69.6
Ig heavy chain VIII region GAL ↓	0.739	0.031	0.0905	87.1	70.9
Ig heavy chain VIII region KOL ↑	0.786	0.028	0.7115	70.9	86.5
Ig heavy chain VIII region NIE ↑	0.794	0.028	0.7700	68.9	92.3
Ig heavy chain VIII region TIL ↓	0.806	0.027	0.1745	82.6	78.4
Ig kappa chain C region ↑	0.777	0.027	16.5040	89.2	60.0
Ig kappa chain VI region EU ↓	0.708	0.032	0.1000	81.9	68.9
Ig kappa chain VI region Roy ↓	0.767	0.028	0.0275	69.0	83.8
Ig kappa chain VII region RPMI6410 ↓	0.749	0.030	0.0220	80.0	75.0
Ig kappa chain VIII region B6 ↑	0.781	0.028	0.9290	59.5	92.9
Ig kappa chain VIV region Fragment ↓	0.705	0.030	0.0130	41.9	99.3
Ig lambda chain V region 4A ↓	0.842	0.025	0.0300	86.5	81.8
Ig lambda chain VI region NEWM ↑	0.775	0.027	0.4015	66.9	83.9
Ig lambda chain VIV region Hil ↑	0.842	0.024	0.8645	68.9	96.1
Ig lambda-2 chain C regions ↑	0.808	0.026	4.7595	76.4	76.8
Ig mu heavy chain disease protein ↓	0.771	0.028	0.1275	74.2	79.7
*Ion-binding*					
Calcium-dependent secretion activator 2 ↑	0.732	0.030	0.0045	61.5	83.9
*Lipid metabolism*					
Apolipoprotein B-100 ↑	0.701	0.030	0.2625	66.2	67.1
Apolipoprotein CIII ↑	0.771	0.028	0.1520	77.0	72.9
Apolipoprotein E ↑	0.701	0.030	0.6435	71.6	63.2
Clusterin ↑	0.719	0.029	0.4350	58.1	80.0
*Nucleic acid modification regulation*					
7-methylguanosine phosphate-specific 5'-nucleotidase ↓	0.803	0.028	0.0050	92.9	70.3
*Protease/protease inhibitor*					
Alpha-2-antiplasmin ↑	0.719	0.030	0.1035	60.1	79.4
Carboxypeptidase B2 ↓	0.773	0.027	0.0025	69.7	81.8
Inter-alpha-trypsin inhibitor heavy chain H4 ↑	0.768	0.027	0.2405	67.6	78.7
*Sialic acid-binding*					
Sialic acid-binding Ig-like lectin 16 ↑	0.728	0.030	0.0125	47.3	95.5
*Transport*					
Afamin ↑	0.744	0.029	0.3470	58.1	87.7
Alpha-2-HS-glycoprotein ↑	0.705	0.030	0.6605	64.9	72.3
Thyroxine-binding globulin ↑	0.720	0.030	0.1015	56.8	83.2
*Unclear or miscellaneous*					
Galectin-3 binding protein ↑	0.774	0.027	0.0655	66.9	78.7
Hepatocyte growth factor-like protein ↑	0.711	0.030	0.0185	55.4	85.2
Leucine-rich alpha-2-glycoprotein ↑	0.755	0.028	0.1785	70.9	72.9
Pigment epithelium-derived factor ↑	0.789	0.026	0.0785	82.4	61.9
Platelet basic protein ↓	0.768	0.027	0.1365	61.3	82.4
Selenoprotein P ↑	0.787	0.027	0.0475	51.4	95.5
Serum amyloid P component ↑	0.731	0.029	0.2655	64.2	78.1
Serum paraoxonase/arylesterase 1 ↓	0.722	0.031	0.1575	58.7	90.5
Small integral membrane protein 23 ↓	0.711	0.030	0.0140	42.6	99.3

Protein content [molecular %; exponentially emPAI/Σ(emPAI) × 100] was used for the protein quantification. Receiver operating characteristic (ROC) analysis [the area under the ROC curve (AUC) >0.7 and p <0.00001] is used to identify proteins that are differentially expressed in HCC. ↑ upregulated in HCC; ↓ downregulated in HCC.

**Table 3:** Differential plasma proteins for cholangiocarcinoma (CCA).

Protein name	AUC	Standard error	Cut-off emPAI%	Sensitivity (%)	Specificity (%)
*Cell-cell interaction or adhesion*					
Lumican ↓	0.743	0.035	0.0840	70.0	76.7
*Coagulation*					
Fibrinogen beta chain ↑	0.800	0.030	4.9850	75.0	69.1
Kininogen-1 ↓	0.713	0.035	0.3105	68.3	71.7
*Complement-associated factors*					
Complement C1r subcomponent ↓	0.738	0.033	0.0595	59.3	80.0
Complement component C8 alpha chain ↓	0.720	0.033	0.1195	63.8	76.7
Complement component C8 beta chain ↓	0.733	0.033	0.0815	59.7	81.7
Properdin ↓	0.742	0.030	0.0165	56.4	85.0
*Immunoglobulin-related protein*					
Ig lambda chain V region 4A ↑	0.711	0.036	0.0255	85.0	53.9
*Lipid metabolism*					
Apolipoprotein A-IV ↓	0.740	0.035	0.6300	49.0	91.7
Apolipoprotein B-100 ↓	0.736	0.037	0.2395	67.9	75.0
Apolipoprotein D ↓	0.802	0.031	0.9495	80.7	70.0
Apolipoprotein M ↓	0.741	0.038	0.0950	76.1	65.0
CD5 antigen-like ↓	0.701	0.037	0.1275	65.4	76.7
Clusterin ↓	0.753	0.036	0.3760	63.4	76.7
Phosphatidylcholine-sterol acyltransferase ↓	0.718	0.036	0.0125	66.3	71.7
Zinc-alpha-2-glycoprotein ↓	0.746	0.035	0.3725	71.2	75.0
*Protease/protease inhibitor*					
Plasma kallikrein ↓	0.794	0.029	0.1200	60.9	90.0
Plasma serine protease inhibitor ↓	0.701	0.033	0.0165	53.5	83.3
Plasminogen ↓	0.763	0.031	0.3765	72.8	71.7
*Transport*					
Afamin ↓	0.758	0.032	0.2010	83.1	61.7
Alpha-2-HS-glycoprotein ↓	0.823	0.027	0.4815	79.8	76.7
Serotransferrin ↓	0.744	0.035	5.0860	61.7	75.0
Vitamin D-binding protein ↓	0.717	0.037	0.4985	83.5	53.3
*Unclear or miscellaneous*					
Gelsolin ↓	0.811	0.030	0.1095	81.1	71.7
Hepatocyte growth factor-like protein ↓	0.706	0.035	0.0105	69.1	65.0

Protein content [molecular %; exponentially emPAI/Σ(emPAI) × 100] was used for the protein quantification. Receiver operating characteristic (ROC) analysis [the area under the ROC curve (AUC) >0.7 and p <0.00001] is used to identify proteins that are differentially expressed in CCA. ↑ upregulated in CCA; ↓ downregulated in CCA.

### Tumor markers with HCC and CCA progression

Regarding the relevance of these biomarker candidates in different tumor stages, we observed that the protein contents of 13 HCC markers, of which 11 were upregulated and two Ig kappa chain-associated proteins were downregulated, varied in different HCC stages (Table 4). We also observed different expression levels of six and two proteins between stages I and II and stages I and III, respectively.

**Table 4:** Differential plasma protein markers in different stages of hepatocellular carcinoma and cholangiocarcinoma.

Variable	Stage I	Stage II	Stage III	Stage IV	ANOVA *P*-value
*Hepatocellular carcinoma*					
2-hydroxyacylsphingosine 1-beta-galactosyltransferas^†^	0.011 ± 0.009	0.017 ± 0.010	0.012 ± 0.009	0.013 ± 0.006	0.015
Afamin^†^	0.326 ± 0.139	0.457 ± 0.224	0.354 ± 0.141	0.352 ± 0.153	0.001
Alpha-2-antiplasmin^†^	0.105 ± 0.045	0.133 ± 0.053	0.118 ± 0.037	0.133 ± 0.052	0.016
Apolipoprotein CIII	0.244 ± 0.190	0.329 ± 0.184	0.227 ± 0.151	0.195 ± 0.093	0.024
Clusterin^†^	0.437 ± 0.161	0.534 ± 0.179	0.475 ± 0.148	0.493 ± 0.207	0.023
Complement factor B	0.358 ± 0.126	0.419 ± 0.136	0.418 ± 0.103	0.385 ± 0.128	0.048
Ig kappa chain VII region RPMI6410^‡^	0.043 ± 0.107	0.059 ± 0.185	0.205 ± 0.356	0.083 ± 0.167	0.005
Ig kappa chain VI region EU^‡^	0.286 ± 0.553	0.225 ± 0.735	0.889 ± 1.362	0.114 ± 0.227	0.003
Ig lambda chain VIV region Hil	2.793 ± 3.218	4.731 ± 5.712	2.286 ± 2.276	2.724 ± 3.380	0.032
Kininogen-1	0.371 ± 0.168	0.446 ± 0.158	0.448 ± 0.151	0.356 ± 0.149	0.043
Pigment epithelium-derived factor^†^	0.107 ± 0.051	0.141 ± 0.053	0.114 ± 0.045	0.125 ± 0.055	0.004
Selenoprotein P^†^	0.042 ± 0.026	0.059 ± 0.033	0.048 ± 0.039	0.018 ± 0.009	0.008
Serum amyloid P component	0.303 ± 0.142	0.371 ± 0.152	0.299 ± 0.123	0.289 ± 0.119	0.041
*Cholangiocarcinoma*					
Plasma serine protease inhibitor	0.014 ± 0.015	0.009 ± 0.011	0.004 ± 0.007	0.003 ± 0.006	0.032

Data are mean values of emPAI% ± standard deviation. emPAI, exponentially modified protein abundance index. *P*-values are obtained from one-way analysis of variance with Scheffe posterior comparison.

^†^emPAI% differs between stage I and II.

^‡^emPAI% differs between stage I and III.

Fold changes of the mean emPAI% values of these 13 HCC markers in comparison with the controls were shown in Fig 2C and S2 Table. Most markers peaked in stage II and dropped in stage III with or without a mild rebound in the end stage. Stage II HCC showed strikingly high expressions of 2-hydroxyacylsphingosine 1-beta-galactosyltransferase (5.61×) and Ig lambda chain VIV region Hil (19.83×). No marker was perfectly correlated with HCC progression. Plasma serine protease inhibitor was the sole protein for CCA staging (Table 4), showing reductions of 36%, 61%, 81%, and 85% in stages I, II, III, and the end stage, respectively (Fig 2D and S2 Table).

### Markers for HCC and CCA prognosis

**Fig 3:**
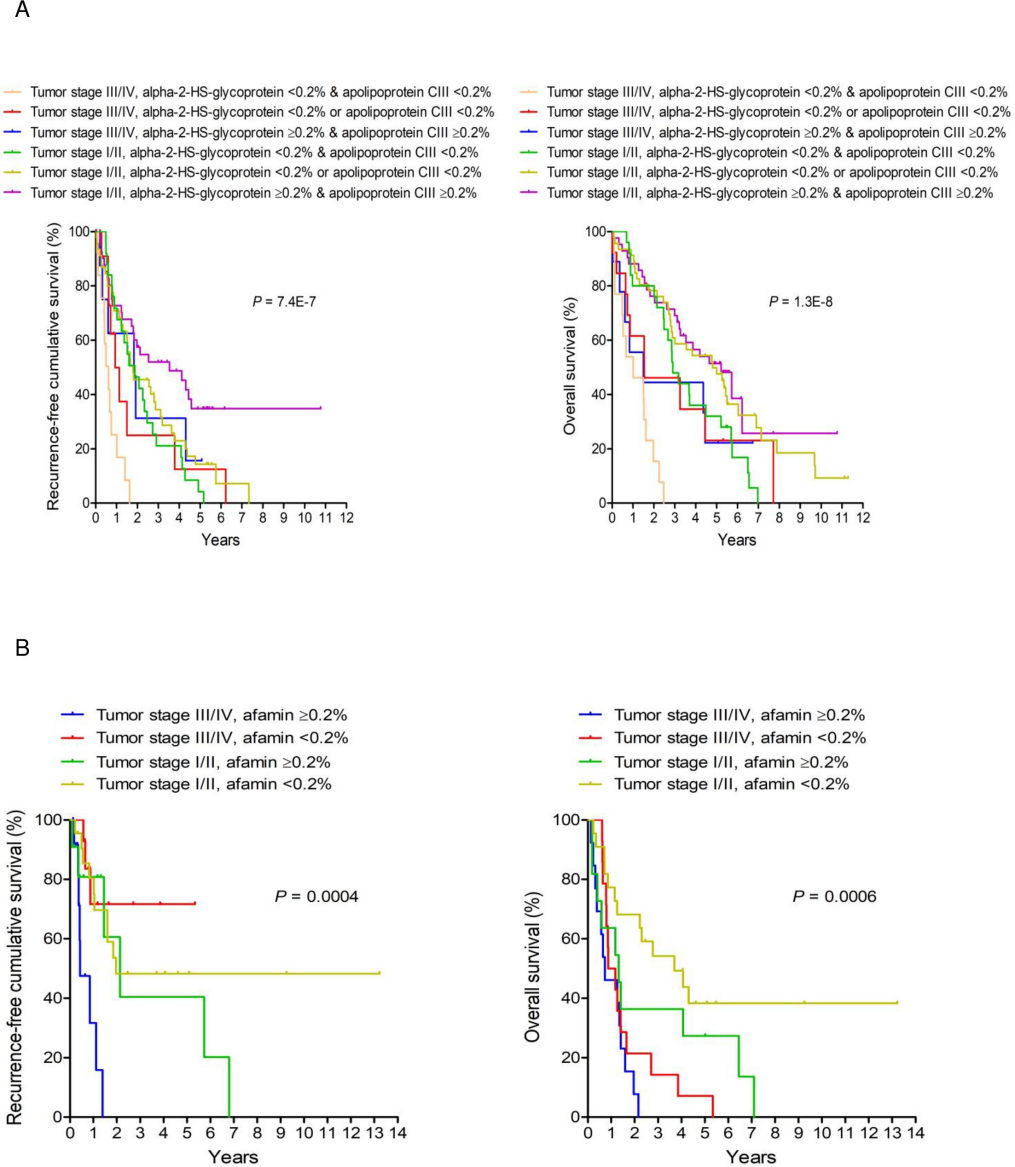
Prognostic analyses of hepatocellular carcinoma (HCC) and cholangiocarcinoma (CCA). Kaplan-Meier curves comparing recurrence-free and overall survivals of (A) HCC patients and (B) CCA patients based on the combination of tumor stage with the prognostic marker panel are shown. *P*-values are obtained from log-rank tests.

Kaplan-Meier analyses revealed six and seven significant markers to evaluate post-surgical tumor recurrence and survival for HCC, respectively, and six and two markers were shown to assess post-surgical tumor recurrence and survival for CCA, respectively. The parameters predicting better recurrence-free and overall survivals in patients with HCC than their counterparts were the values of emPAI% of alpha-2-HS-glycoprotein ≥0.2%, apolipoprotein CIII ≥0.2%, Ig lambda chain VI region NEWM ≥1.0%, and serum amyloid P component ≥0.3% (S3 Fig). Conversely, the presence of Ig heavy chain VIII region CAM in plasma reflected a poor prognosis of HCC. Regarding patients with CCA, a high level of afamin in plasma was correlated with poor outcomes after tumor resection (S4 Fig). Results from stepwise Cox regression analyses showed significant factors that were associated with high recurrence and mortality rates in five years after surgery in patients with HCC (Table 5) or CCA (Table 6). Combination models showed that tumor stage and two prognostic markers, alpha-2-HS-glycoprotein and apolipoprotein CIII, had a similar effect on the prognosis of HCC (Fig 3A). More specifically, HCC stage III or IV and both prognostic markers <0.2% exhibited the poorest post-surgical recurrence-free and overall survivals. By contrast, early-stage HCC and both prognostic markers ≥0.2% demonstrated the most favorable post-surgical outcomes. Furthermore, the level of afamin played a similar role, even more significant than the tumor stage, on the tumor recurrence and overall survival in CCA (Fig 3B).

**Table 5:** Cox regression analyses of hepatocellular carcinoma markers on recurrence and mortality rates in the 5 years after surgery.

	Recurrence				Mortality			
	Univariate		Multivariate		Univariate		Multivariate	
Variable	Hazard ratio (95% CI)	*P*-value	Hazard ratio (95% CI)	*P*-value	Hazard ratio (95% CI)	*P*-value	Hazard ratio (95% CI)	*P*-value
2-hydroxyacylsphingosine 1-beta-galactosyltransferase	0.001 (4.78E-13–9.68E5)	0.498			5.86E-6 (6.64E-16–5.17E4)	0.303	0.211 (0.029–1.537)	0.125
7-methylguanosine phosphate-specific 5'-nucleotidase	2.731 (1.064–7.007)	0.037	1.919 (0.589–6.247)	0.279	2.083 (0.721–6.017)	0.175		
Afamin	0.700 (0.246–1.993)	0.504			0.245 (0.068–0.878)	0.031		
Alpha-2-antiplasmin	0.270 (0.004–17.859)	0.541			0.100 (0.001–8.778)	0.313		
Alpha-2-HS-glycoprotein	0.733 (0.390–1.377)	0.334			0.436 (0.220–0.865)	0.018	1.560 (0.530–4.590)	0.420
Apolipoprotein B-100	0.756 (0.095–5.988)	0.791			0.279 (0.033–2.379)	0.243		
Apolipoprotein CIII	0.303 (0.093–0.979)	0.046	0.328 (0.088–1.225)	0.097	0.307 (0.087–1.075)	0.065		
Apolipoprotein E	1.142 (0.670–1.945)	0.626			0.713 (0.384–1.322)	0.282		
Calcium-dependent secretion activator 2	0.018 (8.48E-20–3.82E15)	0.844			305.228 (2.16E-16–4.32E20)	0.788		
Carboxypeptidase B2	1.07E7 (4.60E-5–2.48E18)	0.225			0.584 (5.10E-14–6.68E12)	0.972		
Clusterin	0.567 (0.185–1.744)	0.323			0.286 (0.082–0.994)	0.049	1.498 (0.235–9.537)	0.669
Coagulation factor XIII A chain	9.58E-12 (3.87E-19–2.37E-4)	0.003	1.62E-12 (1.77E-20–1.47E-4)	0.004	1.27E-13 (2.07E-22–7.82E-5)	0.004	1.24E-14 (1.26E-24–1.22E-4)	0.006
Complement C5	0.191 (0.005–7.415)	0.375			0.142 (0.002–9.287)	0.360		
Complement component C7	1.792 (0.067–47.704)	0.727			1.870 (0.065–53.829)	0.715		
Complement factor B	0.508 (0.109–2.362)	0.388			0.516 (0.099–2.673)	0.430		
Complement factor H-related protein 2	1.041 (0.199–5.434)	0.962			0.331 (0.053–2.081)	0.239		
Fibrinogen alpha chain	0.778 (0.587–1.032)	0.082			0.931 (0.693–1.250)	0.634		
Fibrinogen gamma chain	1.061 (0.848–1.328)	0.604			0.958 (0.746–1.230)	0.734		
Galectin-3 binding protein	7.175 (0.097–533.206)	0.370			0.671 (0.007–63.422)	0.864		
Hepatocyte growth factor-like protein	0.020 (8.04E-8–4.92E3)	0.536			2.50E-4 (1.27E-10–492.019)	0.262		
Ig heavy chain VIII region 23	1.090 (0.720–1.651)	0.683			0.995 (0.607–1.632)	0.986		
Ig heavy chain VIII region CAM	1.082 (0.869–1.347)	0.483			1.029 (0.813–1.303)	0.812		
Ig heavy chain VIII region GAL	0.965 (0.791–1.178)	0.728			0.965 (0.752–1.236)	0.776		
Ig heavy chain VIII region KOL	0.954 (0.874–1.041)	0.294			0.920 (0.833–1.016)	0.101		
Ig heavy chain VIII region NIE	0.950 (0.868–1.039)	0.262			0.922 (0.836–1.018)	0.107		
Ig heavy chain VIII region TIL	1.280 (0.833–1.967)	0.261			1.452 (0.968–2.178)	0.071		
Ig heavy chain VII region ARH77	1.510 (0.068–33.675)	0.795			0.460 (0.010–20.569)	0.689		
Ig heavy chain VI region V35	1.425 (0.702–2.895)	0.327			1.289 (0.637–2.611)	0.480		
Ig kappa chain C region	0.995 (0.980–1.011)	0.556			1.002 (0.985–1.019)	0.843		
Ig kappa chain VIII region B6	0.989 (0.921–1.062)	0.761			0.998 (0.925–1.077)	0.967		
Ig kappa chain VII region RPMI6410*	6.683 (2.530–17.654)	<0.001	3.200 (0.900–11.375)	0.072	7.733 (3.348–17.865)	<0.001	1.977 (0.589–6.637)	0.270
			3.720 (1.168–11.851)	0.026			2.435 (0.774–7.665)	.0128
Ig kappa chain VI region EU*	1.579 (1.214–2.054)	<0.001	1.365 (0.972–1.915)	0.072	1.781 (1.425–2.227)	<0.001	1.500 (1.076–2.090)	0.017
			1.344 (0.975–1.852)	0.071			1.444 (1.062–1.963)	0.019
Ig kappa chain VI region Roy	1.282 (0.539–3.051)	0.574			1.526 (0.659–3.533)	0.323		
Ig kappa chain VIV region Fragment	1.30E-6 (1.55E-19–1.09E7)	0.372			1.41E-6 (4.52E-24–4.41E11)	0.512		
Ig lambda-2 chain C regions	0.989 (0.953–1.027)	0.579			0.987 (0.946–1.029)	0.535		
Ig lambda chain VI region NEWM	0.944 (0.856–1.042)	0.252			0.908 (0.809–1.020)	0.104		
Ig lambda chain VIV region Hil	0.967 (0.923–1.013)	0.160			0.923 (0.863–0.987)	0.019	1.007 (0.942–1.076)	0.839
Ig lambda chain V region 4A	2.354 (0.171–32.449)	0.522			22.147 (3.000–163.510)	0.002	2.159 (0.209–22.306)	0.518
Ig mu heavy chain disease protein	1.378 (0.819–2.319)	0.227			1.459 (0.872–2.440)	0.150		
Inter-alpha-trypsin inhibitor heavy chain H4	0.079 (0.006–0.958)	0.046	1.697 (0.054–53.606)	0.764	0.086 (0.005–1.354)	0.081		
Kininogen-1	0.666 (0.210–2.109)	0.489			0.240 (0.065–0.882)	0.032	1.688 (0.198–14.364)	0.632
Leucine-rich alpha-2-glycoprotein	1.293 (0.224–7.484)	0.774			1.195 (0.183–7.798)	0.853		
Pigment epithelium-derived factor	0.195 (0.004–8.789)	0.400			0.490 (0.010–23.922)	0.719		
Platelet basic protein	0.495 (0.047–5.191)	0.558			0.236 (0.016–3.479)	0.293		
Platelet factor 4	0.002 (1.07E-19–2.28E13)	0.734			1.88E-25 (2.15E-99–1.64E49)	0.512		
Selenoprotein P	0.732 (0.002–336.859)	0.921			0.001 (1.78E-6–1.048)	0.052		
Serum amyloid P component*	0.197 (0.052–0.739)	0.016	1.122 (0.161–7.811)	0.907	0.067 (0.014–0.313)	<0.001	0.131 (0.015–1.174)	0.069
							0.143 (0.029–0.706)	0.017
Serum paraoxonase/arylesterase 1	0.554 (0.015–20.351)	0.748			1.604 (0.040–63.792)	0.802		
Sialic acid-binding Ig-like lectin 16	9.263 (4.36E-7–1.96E8)	0.796			0.001 (5.82E-12–1.24E5)	0.461		
Small integral membrane protein 23	8.66E46 (1.26E18–5.98E75)	0.001	5.80E54 (3.30E24–1.02E85)	<0.001	1.94E-40 (3.43E-159–1.10E79)	0.512		
Thrombospondin 1	1.19E-9 (4.77E-18–0.288)	0.037	1.26E-7 (1.20E-16–131.023)	0.134	341E-4 (3.99E-12–2.92E4)	0.392		
Thyroxine-binding globulin	0.997 (0.024–41.878)	0.999			0.123 (0.002–8.053)	0.326		
Transketolase-like protein 2	2.22E-4 (4.63E-16–1.07E8)	0.540			2.53E4 (1.11E-7–5.76E15)	0.447		

CI, confidence interval.

*Factors are selected into the multivariate analysis after a post-univariate Bonferroni correction (data are shown underlined).

**Table 6:** Cox regression analyses of cholangiocarcinoma markers on recurrence and mortality rates in the 5 years after surgery.

	Recurrence				Mortality			
	Univariate		Multivariate		Univariate		Multivariate	
Variable	Hazard ratio (95% CI)	*P*-value	Hazard ratio (95% CI)	*P*-value	Hazard ratio (95% CI)	*P*-value	Hazard ratio (95% CI)	*P*-value
Afamin	91.311 (1.853–4.50E3)	0.023	8.744 (0.016–4.79E3)	0.500	22.631 (1.113–460.365)	0.042	18.171 (0.988–334.037)	0.051
Alpha-2-HS-glycoprotein	7.614 (1.333–43.499)	0.022	2.090 (0.097–45.097)	0.638	2.159 (0.574–8.123)	0.255		
Apolipoprotein A-IV	0.413 (0.065–2.616)	0.348			0.546 (0.142–2.096)	0.378		
Apolipoprotein B-100	123.094 (2.847–5.32E3)	0.012	8.143 (0.013–5.02E3)	0.522	6.081 (0.379–97.621)	0.202		
Apolipoprotein D	1.269 (0.533–3.025)	0.591			1.436 (0.764–2.700)	0.261		
Apolipoprotein M	10.283 (0.054–1.97E3)	0.385			1.945 (0.045–84.130)	0.729		
CD5 antigen-like	64.235 (0.385–1.07E4)	0.111			1.190 (0.022–64.103)	0.932		
Clusterin	6.619 (0.455–96.306)	0.167			2.039 (0.252–16.464)	0.504		
Complement C1r subcomponent	1.94E5 (5.13E-2–7.34E11)	0.115			39.636 (0.001–2.01E6)	0.506		
Complement component C8 alpha chain	193.889 (0.244–1.54E5)	0.122			14.019 (0.094–2.09E3)	0.301		
Complement component C8 beta chain	483.469 (0.070–3.32E6)	0.170			14.942 (0.015–1.45E4)	0.441		
Fibrinogen beta chain	1.023 (0.954–1.097)	0.519			1.024 (0.976–1.075)	0.330		
Gelsolin	0.998 (9.76E-5–1.02E4)	1.000			0.135 (1.20E-04–151.105)	0.576		
Hepatocyte growth factor-like protein	8.17E15 (0.068–9.75E32)	0.068			1.45E12 (0.022–9.66E25)	0.085		
Ig lambda chain V region 4A	0.603 (0.029–12.407)	0.743			1.836 (0.302–11.145)	0.509		
Kininogen-1	58.563 (2.460–1.39E3)	0.012	2.653 (0.003–2.19E3)	0.776	6.276 (0.628–62.674)	0.118		
Lumican	30.569 (0.027–3.43E4)	0.340			2.773 (0.013–572.137)	0.708		
Phosphatidylcholine-sterol acyltransferase	4.32E-7 (3.19E-23–5.84E9)	0.439			0.005 (1.39E-14–1.77E9)	0.696		
Plasma kallikrein	118.358 (0.159–8.83E4)	0.157			0.350 (0.001–100.757)	0.716		
Plasma serine protease inhibitor	2.88E-11 (1.17E-28–7.08E6)	0.235			1.98E-14 (8.16E-28–0.481)	0.045	2.93E-14 (8.82E-28–0.970)	0.0498
Plasminogen	25.131 (1.564–403.852)	0.023	0.154 (3.49E-4–67.569)	0.546	3.872 (0.499–30.056)	0.195		
Properdin	7.92E-11 (3.79E-31–1.66E10)	0.330			0.834 (2.80E-14–2.48E13)	0.991		
Serotransferrin	1.177 (0.935–1.483)	0.166			0.963 (0.807–1.148)	0.672		
Vitamin D-binding protein	9.025 (1.672–48.697)	0.011	4.632 (0.197–109.078)	0.342	2.337 (0.704–7.752)	0.165		
Zinc-alpha-2-glycoprotein	0.645 (0.054–7.668)	0.728			1.522 (0.292–7.944)	0.618		

CI, confidence interval. No factors are selected into the multivariate analysis by a post-univariate Bonferroni correction.

## Discussion

Current, HCC and CCA are screened and staged mainly using imaging (e.g. ultrasound, X-rays, computed tomography scan, magnetic resonance imaging), pathological tests, and laboratory tests. However, a portion of patients with HCC or CCA are misdiagnosed or receive a delayed diagnosis and are typically amenable to undergo surgical resection or liver transplantation. Moreover, annually, nearly 1 million liver cancer-related deaths continue to be reported worldwide. This information indicated the urgency of developing novel highly sensitive and specific screening platforms, by overcoming bottlenecks of traditional systems, for the determination of early onset of hepatobiliary cancers and manifestations of their deterioration.

Label-free quantitative mass spectrometry is applicable to a wide variety of research fields, for which the following three approaches are the most commonly used: spectral counting, peptide chromatographic peak area, and emPAI. Spectral counting method measures the number of MS/MS spectra of a given protein. The peak area method involves calculating and comparing the mean intensity of peak areas for all peptides from each protein [10,11]. The emPAI quantification method is a modified form of spectral counting [7]. Dowle et al. compared the diagnostic accuracy of the three label-free methods by analyzing a solution of 18 exogenous proteins added to *E*. *coli* lysate and found that spectral counting and emPAI displayed satisfactory positive predictive values for Gross differences (1.5- to ∞-fold difference), whereas peak area performed best for smaller-fold differences (1.1- to 1.75-fold) [12]. A study using complex mixtures of mouse neuro2A cells demonstrated that emPAI was strongly correlated with the actual protein amount in a wide dynamic range from 30 fmol/μL to 1.8 pmol/μL in the sample solution [7]. Nevertheless, this method is more suitable for interprotein and intrasample quantification with its intersample performance showing little evidence [13–16]. Shinoda et al. used emPAI% to calculate the molecular percentage of all the identified proteins for fractionated samples in a large-scale LC-MS/MS analysis [8]. We adopted emPAI% using a fixed amount of protein for each sample in spite of the fact that absolute protein levels have yet to be known to perform an LC-MS/MS-based intersample, comparative proteomic study.

Our previous study enrolled patients with HCC, CCA, or combined hepatocellular-cholangiocarcinoma, in which a protein biomarker pool containing 57 entities to discriminate the patients from the controls was established and the clinical relevance of different glycosylation patterns on complement C3 in HCC was subsequently analyzed [6]. Although using the same dataset, we used a different strategy here to identify specific plasma biomarkers for HCC or CCA. In addition, only albumin- and IgG-depleted sample fraction rather than whole plasma proteins was used in the mass spectrometry analysis in this study to reduce the interference caused by albumin and IgG. Moreover, simplified sample processing may improve the feasibility of applying these tumor biomarkers in clinical testing.

There have been numerous proteomic studies and diverse tumor biomarker candidates for liver cancer reported. Awan et al. concluded 38 liver-specific secreted or shed protein marker candidates for HCC from seven publicly accessible gene and protein databases [17]. Also, Kimhofer et al. reviewed 22 reports and selected 29 protein biomarkers for HCC [18]. Only one (apolipoprotein CIII) and four (afamin, apolipoprotein B-100, clusterin, and serum paraoxonase/arylesterase 1) common HCC biomarkers were identified when comparing our data to these reviews, respectively. Moreover, haptoglobin is the only common HCC biomarker between these two review papers. From our plasma proteomics, several Ig-related proteins or non-liver-specific proteins were linked to HCC or CCA. It is not surprising to see few common HCC biomarkers among different reports and identify many novel biomarkers in our study because of different sample types, subject enrollment criteria, sample collection, processing procedures, analytic approaches, and biomarker selection criteria from different works of literature.

Combined hepatocellular-cholangiocarcinoma was not included because the number of patients (2 in stage I, 5 in stage II, 3 in stage III, and 2 in stage IV) was too small to acquire precise statistical results. Moreover, we did not include complement C3 on the list of HCC markers in this analytic model. Intriguingly, no ideal markers that perfectly correlated with HCC progression were found. The identified 13 HCC biomarker candidates fluctuated in different tumor stages, and their quantities did not perfectly correlate with the disease severity. The result of the emPAI% calculation shows that these markers are not tumor-specific proteins but possess distinguishable levels during hepatocarcinogenesis. We observed instead that most of them increased from stage I to II, reduced in stage III, and finished with or without a rebound in the end stage. A similar result was noted using a nonparametric statistical model. Although detecting enhanced levels of these biomarkers resulting from the growth of a solitary tumor from stage I to II with affordable liver functions is plausible, when transitioning to stage III, liver functions might be severely impaired because of expansions of multiple large tumors, thereby contributing to a detrimental effect of protein synthesis. Stage III might see limited production and secretion of biomarker proteins by deep cancerous cells because of central tumor necrosis, a typical feature of advanced HCC. In the final stage, the abundance of biomarker proteins is dependent on the number and size of tumor foci that metastasize to regional lymph nodes or distal organs. These circulating proteins may serve as markers may reflect not only the progression of liver cancer but also the physiological condition of the liver system in patients. One of the limitations of this study is that we could not enroll many patients with late-stage HCC or CCA since only a few patients with advanced-stage HCC or CCA are amenable to undergo surgery. Nonetheless, we found that some of the biomarker candidates decreased in late-stage HCC and identified some biomarker candidates that specifically changed in advanced or metastatic HCC or CCA. These findings, even though they were obtained from a relatively small number of patients, provide significant information as well about the practicability of using these circulating biomarkers to non-invasively evaluate the status and progression of HCC or CCA.

Apolipoprotein C-III and serum amyloid P component are another two biomarkers associated with a better prognosis of HCC. Apolipoprotein C-III is a key component in the regulation of triacylglycerol-rich lipoproteins and high-density lipoproteins [19,20], whereas serum amyloid P component, of the pentraxin superfamily, is an acute-phase reactant produced in the liver that activates the classical complement pathway [21,22]. They reflect the capability, at least partially, of the liver on the lipid metabolism and immune activation. Stage-specific analyses revealed that these two proteins showed maximum expression in stage II HCC and continuously reduction afterward, suggesting a decline of liver and immune functions by late-stage HCC. Therefore, not surprising to see a correspondence of low levels of apolipoprotein C-III and serum amyloid P component with a high post-surgical recurrent rate and short survival period in patients with HCC.

Plasma serine protease inhibitor (SERPINA5), mainly synthesized in the liver, is a multifunctional tumor suppressor that reduces the metastatic property of hepatic cancer cell lines by disrupting the fibronectin–integrin signaling pathway [23–25]. Repression of this protein has been reported in several cancer types, such as prostate, kidney, ovary, and liver [25–29]. Our result also corroborated this finding and demonstrated a downregulation of plasma serine protease inhibitor in patients with CCA, with a higher level observed in non-metastatic than metastatic CCA. Unlike the trend of HCC biomarker proteins in different tumor stages, plasma serine protease inhibitor decreased gradually with CCA progression. Moreover, it gives impetus to a better 5-year survival of patients with CCA. In contrast to plasma serine protease inhibitor, afamin, a fellow of albumin, a-fetoprotein, and vitamin D-binding protein family [30], seems to be unconducive for the post-surgical outcome of CCA. A low afamin level in serum has been reported in ovarian cancer and CCA [31,32]. We currently do not know the physiological roles of plasma serine protease inhibitor and afamin in cholangiocytes. Future researches should focus on clarifying the molecular mechanisms and pathogenic effects of these two proteins.

## Conclusion

Our large-scale label-free, quantitative plasma proteome study identified significant stage-specific and prognostic biomarkers for HCC and CCA. Our findings may provide new insight into clinical application startups using these biomarkers in the diagnosis and follow-up of hepatobiliary cancers. From a clinical perspective, we expect proteomics of liquid biopsy to be added to routine laboratory testing for clinical oncology soon.

## Supporting information

S1 Table Characteristics of patients with stage IIIA to IIIC hepatocellular carcinoma. (DOCX)

S2 Table Fold change of tumor markers in different tumor stages. (DOCX)

S1 Fig Comparisons of circulating or secretory protein biomarkers for hepatocellular carcinoma (HCC). A Venn diagram for circulating or secretory protein biomarker candidates for HCC that were identified in the present study and reported in two review articles is shown.
(PPTX)

S2 Fig Comparisons of protein contents in different status of hepatocellular carcinoma (HCC) or cholangiocarcinoma (CCA). Values of the percentage of exponentially modified protein abundance index (emPAI%) of protein markers between advanced-stage and non-advanced-stage HCC (A) and CCA (C) as well as between metastatic and non-metastatic HCC (B) and CCA (D) are shown in Tukey box-and-whisker plots. *P*-values are obtained from Mann–Whitney *U* tests.
(PPTX)

S3 Fig Relevance of biomarkers in the prognosis of hepatocellular carcinoma. Kaplan-Meier analyses of associations between different hepatocellular carcinoma markers with (A) recurrence-free survival and (B) overall survival in the patients (n = 148) are shown. *P*-values are obtained from log-rank tests.
(PPTX)

S4 Fig Relevance of biomarkers in the prognosis of cholangiocarcinoma. Kaplan-Meier analyses of associations between different cholangiocarcinoma markers with (A) recurrence-free survival and (B) overall survival in the patients (n = 60) are shown. *P*-values are obtained from log-rank tests.
(PPTX)

## Abbreviations

CCA: cholangiocarcinoma
CI: confidence interval
emPAI: exponentially modified protein abundance index
HCC: hepatocellular carcinoma
LC-MS/MS: liquid chromatography–tandem mass spectrometry. Standard abbreviations not requiring definition can be found in *The AMA Manual of Style*

## References

[1] de Martel C, Georges D, Bray F, Ferlay J, Clifford GM. Global burden of cancer attributable to infections in 2018: a worldwide incidence analysis. The Lancet Global health. 2020;8(2):e180–e90. 10.1016/S2214-109X(19)30488-7 . 31862245

[2] Raza A, Sood GK. Hepatocellular carcinoma review: current treatment, and evidence-based medicine. World journal of gastroenterology. 2014;20(15):4115–27. 10.3748/wjg.v20.i15.4115 24764650 PMC3989948

[3] Tella SH, Kommalapati A, Borad MJ, Mahipal A. Second-line therapies in advanced biliary tract cancers. The Lancet Oncology. 2020;21(1):e29–e41. 10.1016/S1470-2045(19)30733-8 . 31908303

[4] Brivio S, Cadamuro M, Strazzabosco M, Fabris L. Tumor reactive stroma in cholangiocarcinoma: The fuel behind cancer aggressiveness. World journal of hepatology. 2017;9(9):455–68. 10.4254/wjh.v9.i9.455 28396716 PMC5368623

[5] Labgaa I, Craig AJ, Villanueva A. Diagnostic and Prognostic Performance of Liquid Biopsy in Hepatocellular Carcinoma Liquid Biopsy in Cancer Patients: Springer; 2017 p. 125–35.

[6] Chang TT, Cheng JH, Tsai HW, Young KC, Hsieh SY, Ho CH. Plasma proteome plus site-specific N-glycoprofiling for hepatobiliary carcinomas. The journal of pathology Clinical research. 2019;5(3):199–212. 10.1002/cjp2.136 31136099 PMC6648390

[7] Ishihama Y, Oda Y, Tabata T, Sato T, Nagasu T, Rappsilber J, et al Exponentially modified protein abundance index (emPAI) for estimation of absolute protein amount in proteomics by the number of sequenced peptides per protein. Molecular & cellular proteomics: MCP. 2005;4(9):1265–72. 10.1074/mcp.M500061-MCP200 . 15958392

[8] Shinoda K, Tomita M, Ishihama Y. emPAI Calc—for the estimation of protein abundance from large-scale identification data by liquid chromatography-tandem mass spectrometry. Bioinformatics. 2010;26(4):576–7. 10.1093/bioinformatics/btp700 . 20031975

[9] Heberle H, Meirelles GV, da Silva FR, Telles GP, Minghim R. InteractiVenn: a web-based tool for the analysis of sets through Venn diagrams. BMC bioinformatics. 2015;16:169 10.1186/s12859-015-0611-3 . 25994840 PMC4455604

[10] Liu H, Sadygov RG, Yates JR 3rd. A model for random sampling and estimation of relative protein abundance in shotgun proteomics. Analytical chemistry. 2004;76(14):4193–201. 10.1021/ac0498563 . 15253663

[11] Chelius D, Bondarenko PV. Quantitative profiling of proteins in complex mixtures using liquid chromatography and mass spectrometry. Journal of proteome research. 2002;1(4):317–23. 10.1021/pr025517j . 12645887

[12] Dowle AA, Wilson J, Thomas JR. Comparing the Diagnostic Classification Accuracy of iTRAQ, Peak-Area, Spectral-Counting, and emPAI Methods for Relative Quantification in Expression Proteomics. Journal of proteome research. 2016;15(10):3550–62. 10.1021/acs.jproteome.6b00308 . 27546623

[13] Urbani A, Sirolli V, Lupisella S, Levi-Mortera S, Pavone B, Pieroni L, et al Proteomic investigations on the effect of different membrane materials on blood protein adsorption during haemodialysis. Blood transfusion = Trasfusione del sangue. 2012;10 Suppl 2:s101–12. 10.2450/2012.014S 22890260 PMC3418624

[14] Ternan NG, Jain S, Graham RL, McMullan G. Semiquantitative analysis of clinical heat stress in Clostridium difficile strain 630 using a GeLC/MS workflow with emPAI quantitation. PloS one. 2014;9(2):e88960 10.1371/journal.pone.0088960 24586458 PMC3933415

[15] Carvalhais V, Cerca N, Vilanova M, Vitorino R. Proteomic profile of dormancy within Staphylococcus epidermidis biofilms using iTRAQ and label-free strategies. Applied microbiology and biotechnology. 2015;99(6):2751–62. 10.1007/s00253-015-6434-3 . 25672847

[16] Fish-Low CY, Than LTL, Ling KH, Lin Q, Sekawi Z. Plasma proteome profiling reveals differentially expressed lipopolysaccharide-binding protein among leptospirosis patients. Journal of microbiology, immunology, and infection = Wei mian yu gan ran za zhi. 2019 10.1016/j.jmii.2018.12.015 . 31029530

[17] Awan FM, Naz A, Obaid A, Ali A, Ahmad J, Anjum S, et al Identification of Circulating Biomarker Candidates for Hepatocellular Carcinoma (HCC): An Integrated Prioritization Approach. PloS one. 2015;10(9):e0138913 10.1371/journal.pone.0138913 . 26414287 PMC4586137

[18] Kimhofer T, Fye H, Taylor-Robinson S, Thursz M, Holmes E. Proteomic and metabonomic biomarkers for hepatocellular carcinoma: a comprehensive review. British journal of cancer. 2015;112(7):1141–56. 10.1038/bjc.2015.38 . 25826224 PMC4385954

[19] Breyer ED, Le NA, Li X, Martinson D, Brown WV. Apolipoprotein C-III displacement of apolipoprotein E from VLDL: effect of particle size. Journal of lipid research. 1999;40(10):1875–82. . 10508207

[20] Pavlic M, Valero R, Duez H, Xiao C, Szeto L, Patterson BW, et al Triglyceride-rich lipoprotein-associated apolipoprotein C-III production is stimulated by plasma free fatty acids in humans. Arteriosclerosis, thrombosis, and vascular biology. 2008;28(9):1660–5. 10.1161/ATVBAHA.108.169383 18556566 PMC3750732

[21] Hicks PS, Saunero-Nava L, Du Clos TW, Mold C. Serum amyloid P component binds to histones and activates the classical complement pathway. Journal of immunology. 1992;149(11):3689–94. . 1431140

[22] Bottazzi B, Inforzato A, Messa M, Barbagallo M, Magrini E, Garlanda C, et al The pentraxins PTX3 and SAP in innate immunity, regulation of inflammation and tissue remodelling. Journal of hepatology. 2016;64(6):1416–27. 10.1016/j.jhep.2016.02.029 26921689 PMC5414834

[23] Francis RB Jr., Thomas W. Behaviour of protein C inhibitor in intravascular coagulation and liver disease. Thrombosis and haemostasis. 1984;52(1):71–4. . 6548588

[24] Sil H, Sen T, Chatterjee A. Fibronectin-integrin (alpha5beta1) modulates migration and invasion of murine melanoma cell line B16F10 by involving MMP-9. Oncology research. 2011;19(7):335–48. 10.3727/096504011x13079697132925 . 21936403

[25] Jing Y, Jia D, Wong CM, Oi-Lin Ng I, Zhang Z, Liu L, et al SERPINA5 inhibits tumor cell migration by modulating the fibronectin-integrin beta1 signaling pathway in hepatocellular carcinoma. Molecular oncology. 2014;8(2):366–77. 10.1016/j.molonc.2013.12.003 24388360 PMC5528558

[26] Cao Y, Becker C, Lundwall A, Christensson A, Gadaleanu V, Lilja H, et al Expression of protein C inhibitor (PCI) in benign and malignant prostatic tissues. The Prostate. 2003;57(3):196–204. 10.1002/pros.10296 . 14518028

[27] Wakita T, Hayashi T, Nishioka J, Tamaru H, Akita N, Asanuma K, et al Regulation of carcinoma cell invasion by protein C inhibitor whose expression is decreased in renal cell carcinoma. International journal of cancer. 2004;108(4):516–23. 10.1002/ijc.11594 . 14696115

[28] Asanuma K, Yoshikawa T, Hayashi T, Akita N, Nakagawa N, Hamada Y, et al Protein C inhibitor inhibits breast cancer cell growth, metastasis and angiogenesis independently of its protease inhibitory activity. International journal of cancer. 2007;121(5):955–65. 10.1002/ijc.22773 . 17450526

[29] Bijsmans IT, Smits KM, de Graeff P, Wisman GB, van der Zee AG, Slangen BF, et al Loss of SerpinA5 protein expression is associated with advanced-stage serous ovarian tumors. Modern pathology: an official journal of the United States and Canadian Academy of Pathology, Inc. 2011;24(3):463–70. 10.1038/modpathol.2010.214 . 21102419

[30] Lichenstein HS, Lyons DE, Wurfel MM, Johnson DA, McGinley MD, Leidli JC, et al Afamin is a new member of the albumin, alpha-fetoprotein, and vitamin D-binding protein gene family. The Journal of biological chemistry. 1994;269(27):18149–54. . 7517938

[31] Jackson D, Craven RA, Hutson RC, Graze I, Lueth P, Tonge RP, et al Proteomic profiling identifies afamin as a potential biomarker for ovarian cancer. Clinical cancer research: an official journal of the American Association for Cancer Research. 2007;13(24):7370–9. 10.1158/1078-0432.CCR-07-0747 . 18094419

[32] Tolek A, Wongkham C, Proungvitaya S, Silsirivanit A, Roytrakul S, Khuntikeo N, et al Serum alpha1beta-glycoprotein and afamin ratio as potential diagnostic and prognostic markers in cholangiocarcinoma. Experimental biology and medicine. 2012;237(10):1142–9. 10.1258/ebm.2012.012215 . 23104505

