# Supplementary material for: Plasma proteome atlas for differentiating tumor stage and post-surgical prognosis of hepatocellular carcinoma and cholangiocarcinoma
Source: PLoS One. 2020 Aug 26;15(8):e0238251. doi: 10.1371/journal.pone.0238251 (PMC7449477; doi:10.1371/journal.pone.0238251)
Supplement: S1 Table — (DOCX) [file pone.0238251.s001.docx]

| Supplementary Table 1. Characteristics of patients with stage IIIA to IIIC hepatocellular carcinoma | | | | |
| --- | --- | --- | --- | --- |
| Variable | Stage IIIA (n = 20) | Stage IIIB (n = 5) | Stage IIIC (n = 6) | *P*-value |
| Male, n (%) | 14 (70.0%) | 3 (60.0%) | 4 (66.7%) | 0.911 |
| Age (years) | 63 (44 – 84) | 67 (50 – 73) | 55.5 (46 – 66) | 0.219 |
| Alanine aminotransferase (U/L) | 48 (18 – 410) | 175 (51 – 436) | 33.5 (13 – 73) | 0.053 |
| Aspartate aminotransferase (U/L) | 47.5 (22 – 300) | 68 (41 – 800) | 51.5 (30 – 71) | 0.290 |
| Albumin (g/dL) | 3.6 (2.5 – 4.8) | 4.5 (1.8 – 4.7) | 4.0 (1.9 – 4.4) | 0.573 |
| Alkaline phosphatase (U/L)* | 108 (50 – 976) | 202.5 (54 – 384) | 117.5 (97 – 215) | 0.506 |
| Total bilirubin (mg/dL) | 0.6 (0.3 – 4.4) | 0.7 (0.6 – 7.0) | 0.6 (0.5 – 4.2) | 0.213 |
| α-fetoprotein (ng/mL)* | 6.1 (0.9 – 32420.0) | 2093.0 (857.3 – 15751.0) | 81.0 (60.0 – 725.8) | 0.375 |
| Red blood cell (10^6^/μL) | 4.1 (3.2 – 4.8) | 4.0 (3.9 – 4.3) | 4.3 (2.9 – 4.7) | 0.689 |
| White blood cell (10^3^/μL) | 6.4 (3.9 – 10.4) | 6.8 (4.3 – 9.9) | 5.4 (4.1 – 8.0) | 0.525 |
| Platelet (10^3^/μL) | 209 (88 – 400) | 194 (99 – 332) | 192 (112 – 255) | 0.599 |
| Hepatitis B, n (%) | 13 (65.0%) | 3 (60.0%) | 5 (83.3%) | 0.646 |
| Hepatitis C, n (%) | 4 (20.0%) | 0 (0.0%) | 1 (16.7%) | 0.553 |
| Liver cirrhosis, n (%) | 9 (45.0%) | 4 (80.0%) | 6 (100.0%) | 0.034 |
| Follow-up 5-Year recurrence, n (%) | 15 (75.0%) | 4 (80.0%) | 5 (83.3%) | 0.902 |
| Follow-up 5-Year survivals, n (%) | 5 (25.0%) | 1 (20.0%) | 0 (0.0%) | 0.397 |
| Data are numbers (percentages) or median values (minimum − maximum). *contain missing values. Nominal values are compared using Pearson Chi-square tests. Continuous variables are compared using Kruskal-Wallis tests | | | | |
