# Supplementary material for: Plasma proteome atlas for differentiating tumor stage and post-surgical prognosis of hepatocellular carcinoma and cholangiocarcinoma
Source: PLoS One. 2020 Aug 26;15(8):e0238251. doi: 10.1371/journal.pone.0238251 (PMC7449477; doi:10.1371/journal.pone.0238251)
Supplement: S2 Table — (DOCX) [file pone.0238251.s002.docx]

| Supplementary Table 2. Fold change of tumor markers in different tumor stages | | | | |
| --- | --- | --- | --- | --- |
| Variable | Stage I | Stage II | Stage III | Stage IV |
| *Hepatocellular carcinoma* |  |  |  |  |
| 2-hydroxyacylsphingosine 1-beta-galactosyltransferase | 3.795 | 5.608 | 4.100 | 4.349 |
| Afamin | 1.226 | 1.722 | 1.334 | 1.323 |
| Alpha-2-antiplasmin | 1.302 | 1.652 | 1.457 | 1.647 |
| Apolipoprotein CIII | 1.827 | 2.462 | 1.700 | 1.459 |
| Clusterin | 1.121 | 1.372 | 1.219 | 1.265 |
| Complement factor B | 1.187 | 1.388 | 1.387 | 1.275 |
| Ig kappa chain VII region RPMI6410 | 0.282 | 0.384 | 1.340 | 0.544 |
| Ig kappa chain VI region EU | 0.753 | 0.592 | 2.342 | 0.299 |
| Ig lambda chain VIV region Hil | 11.705 | 19.831 | 9.582 | 11.419 |
| Kininogen-1 | 1.159 | 1.395 | 1.402 | 1.113 |
| Pigment epithelium-derived factor | 1.506 | 1.990 | 1.616 | 1.764 |
| Selenoprotein P | 2.128 | 2.981 | 2.412 | 0.918 |
| Serum amyloid P component | 1.413 | 1.731 | 1.394 | 1.349 |
|  |  |  |  |  |
| *Cholangiocarcinoma* |  |  |  |  |
| Plasma serine protease inhibitor | 0.640 | 0.389 | 0.193 | 0.146 |
| Data are folds of protein content in patients normalized to those in controls. | | | | |
