# Supplementary material for: Plasma proteome atlas for differentiating tumor stage and post-surgical prognosis of hepatocellular carcinoma and cholangiocarcinoma
Source: PLoS One. 2020 Aug 26;15(8):e0238251. doi: 10.1371/journal.pone.0238251 (PMC7449477; doi:10.1371/journal.pone.0238251)
Supplement: S1 Fig — A Venn diagram for circulating or secretory protein biomarker candidates for HCC that were identified in the present study and reported in two review articles is shown. (PPTX) [file pone.0238251.s003.pptx]

## Slide 1
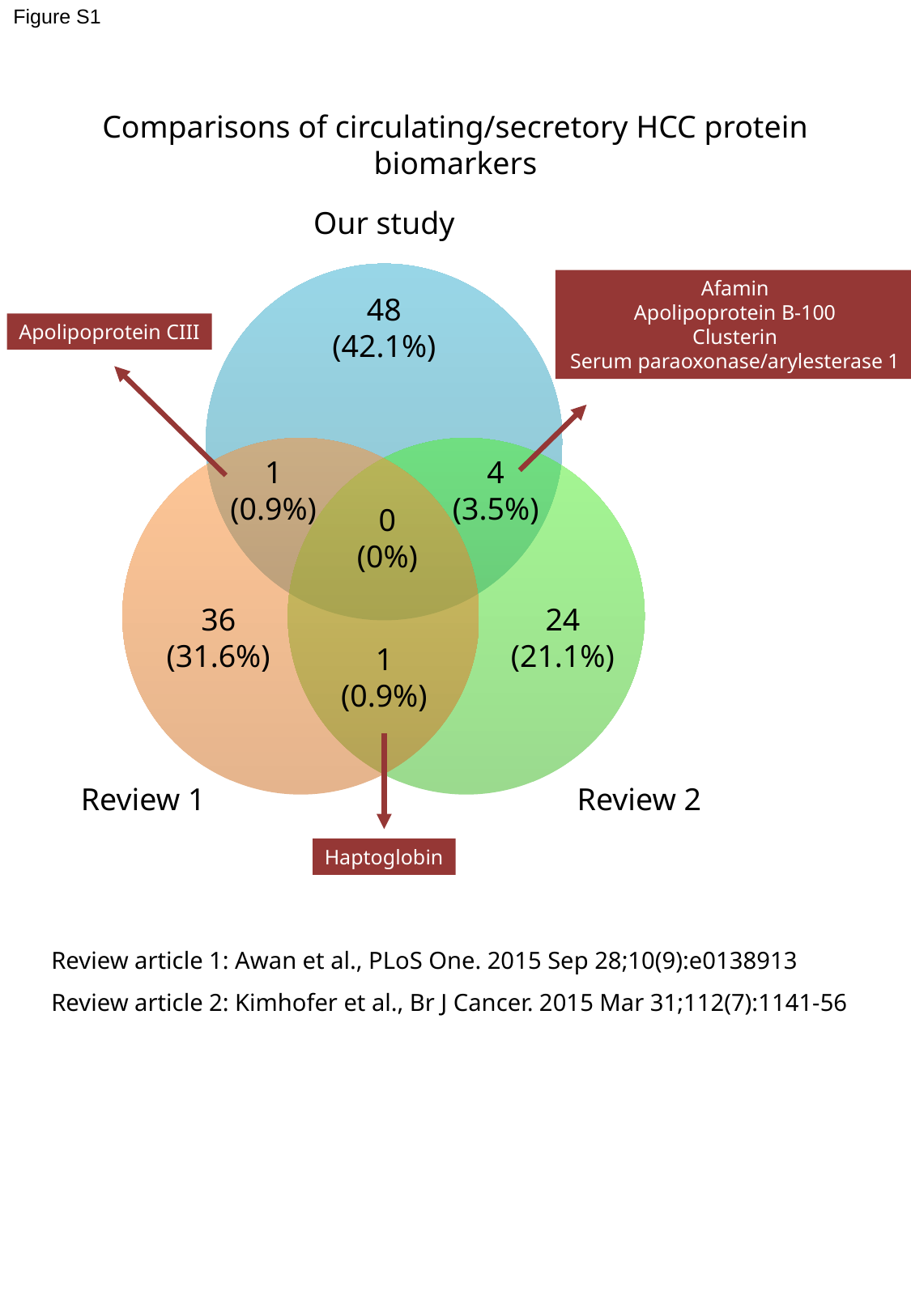

Figure S1
Comparisons of circulating/secretory HCC protein biomarkers
Our study
Afamin
Apolipoprotein B-100
Clusterin
Serum paraoxonase/arylesterase 1
48
(42.1%)
Apolipoprotein CIII
1
(0.9%)
4
(3.5%)
0
(0%)
36
(31.6%)
24
(21.1%)
1
(0.9%)
Review 1
Review 2
Haptoglobin
Review article 1: Awan et al., PLoS One. 2015 Sep 28;10(9):e0138913
Review article 2: Kimhofer et al., Br J Cancer. 2015 Mar 31;112(7):1141-56
