# Supplementary figures and images for: Plasma proteome atlas for differentiating tumor stage and post-surgical prognosis of hepatocellular carcinoma and cholangiocarcinoma
Source: PLoS One. 2020 Aug 26;15(8):e0238251. doi: 10.1371/journal.pone.0238251 (PMC7449477; doi:10.1371/journal.pone.0238251)

## Slide 1
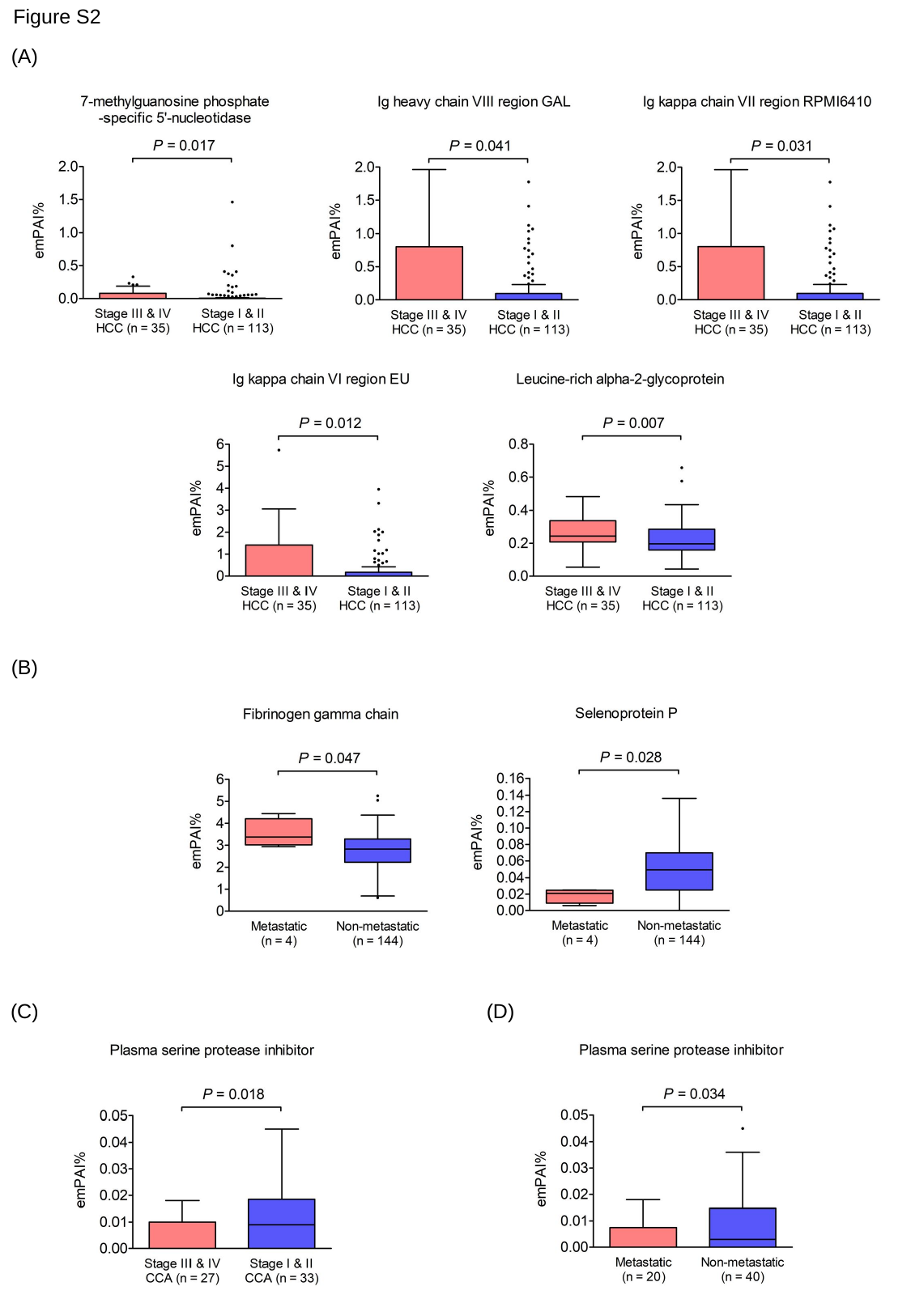

Figure S2
(A)
(B)
(C)
(D)

Supplement: S2 Fig — Values of the percentage of exponentially modified protein abundance index (emPAI%) of protein markers between advanced-stage and non-advanced-stage HCC (A) and CCA (C) as well as between metastatic and non-metastatic HCC (B) and CCA (D) are shown in Tukey box-and-whisker plots. P-values are obtained from Mann–Whitney U tests. (PPTX) [file pone.0238251.s004.pptx]

## Slide 1
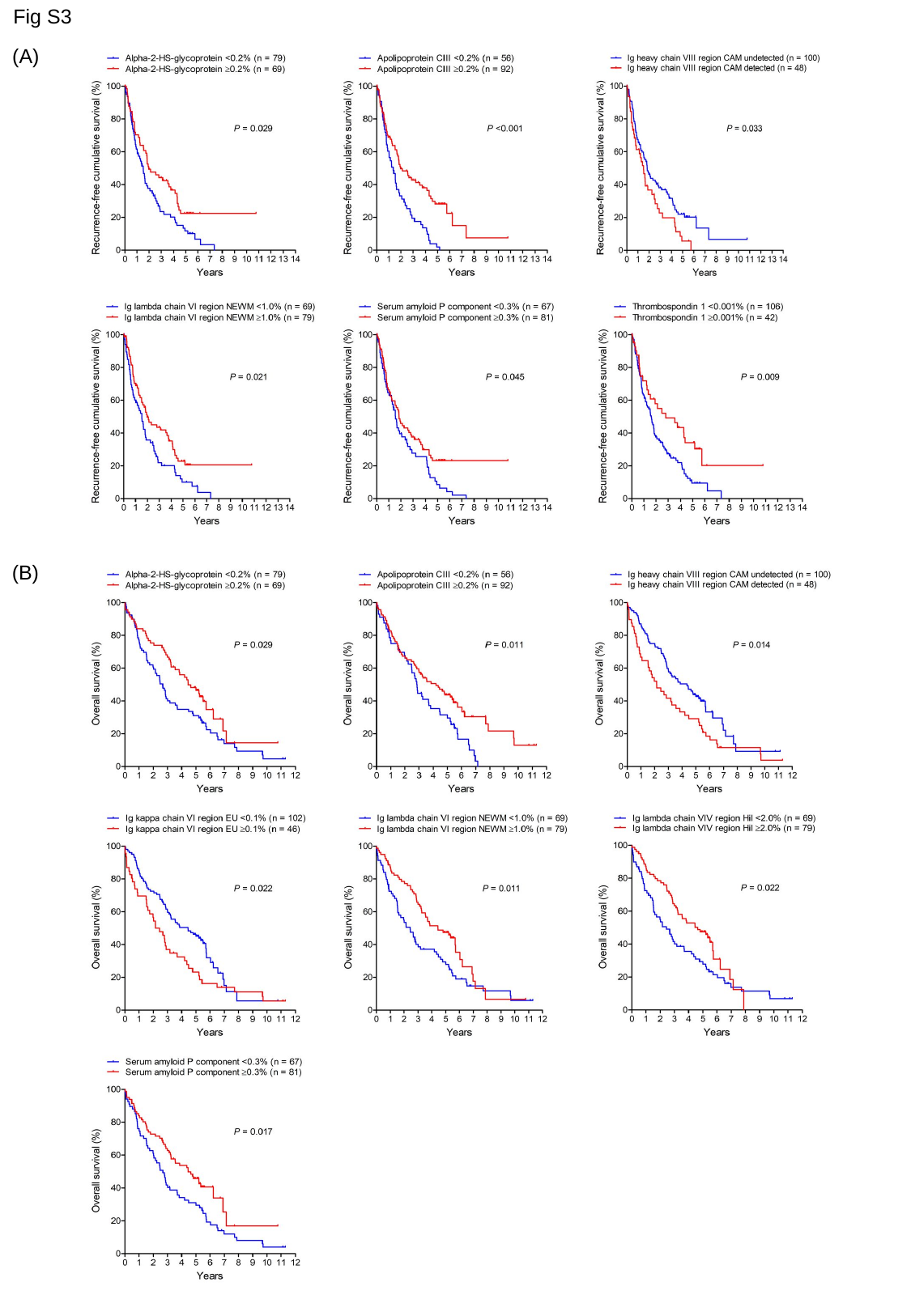

Fig S3
(A)
(B)

Supplement: S3 Fig — Kaplan-Meier analyses of associations between different hepatocellular carcinoma markers with (A) recurrence-free survival and (B) overall survival in the patients (n = 148) are shown. P-values are obtained from log-rank tests. (PPTX) [file pone.0238251.s005.pptx]

## Slide 1
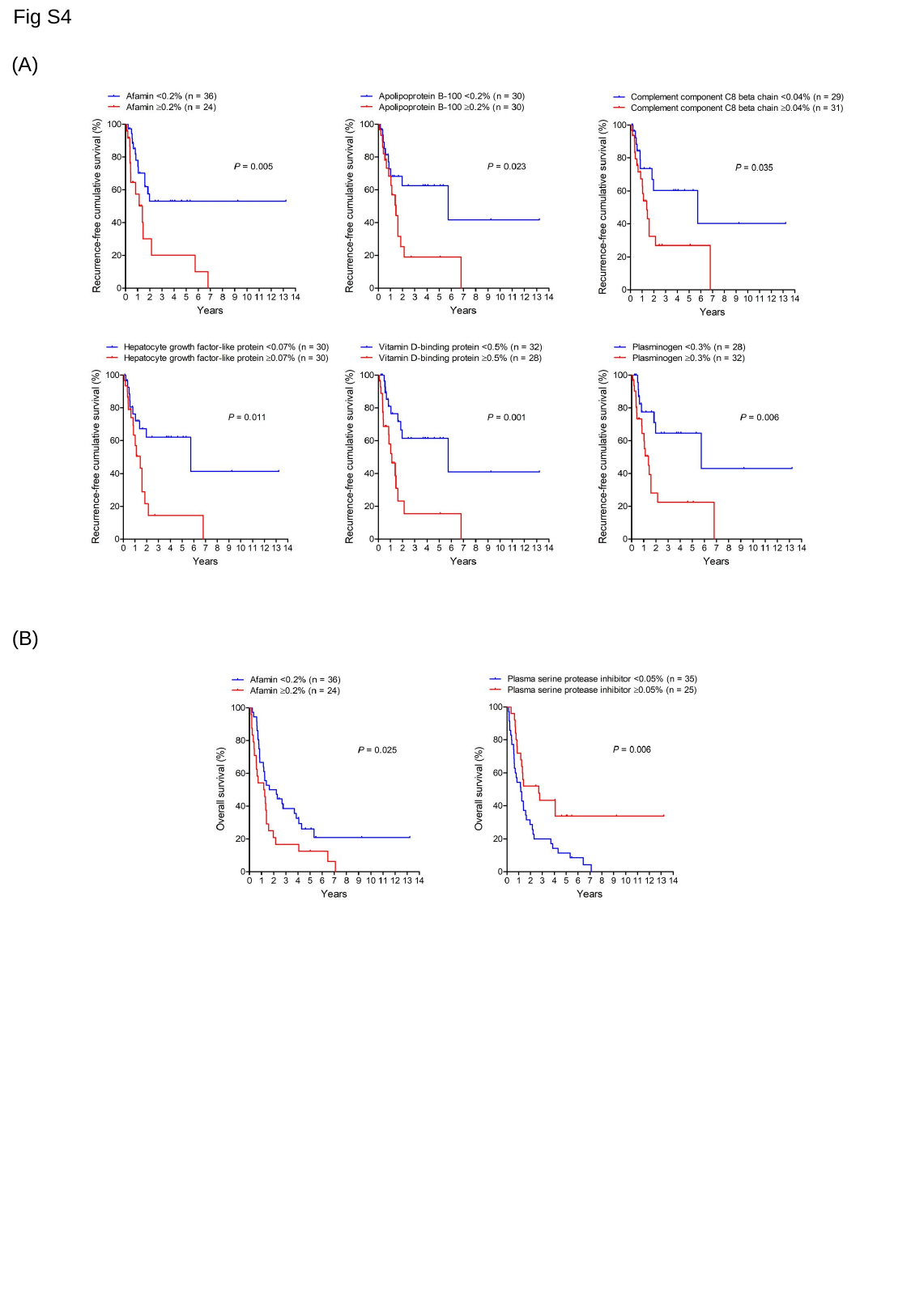

Fig S4
(A)
(B)

Supplement: S4 Fig — Kaplan-Meier analyses of associations between different cholangiocarcinoma markers with (A) recurrence-free survival and (B) overall survival in the patients (n = 60) are shown. P-values are obtained from log-rank tests. (PPTX) [file pone.0238251.s006.pptx]
